# The Role of Notch Signaling Pathway in Non-Alcoholic Fatty Liver Disease

**DOI:** 10.3389/fmolb.2021.792667

**Published:** 2021-11-24

**Authors:** Hao Xu, Lin Wang

**Affiliations:** Department of Hepatobiliary Surgery, Xi-Jing Hospital, The Fourth Military Medical University, Xi’an, China

**Keywords:** Notch signaling pathway, non-alcoholic fatty liver disease (NAFLD), steatohepatitis, lipid metabolism, insulin resistance (IR), fibrogenesis, autophagy

## Abstract

Non-alcoholic fatty liver disease (NAFLD) is the most common chronic liver disease worldwide, and progressive NAFLD can develop into non-alcoholic steatohepatitis (NASH), liver cirrhosis, or hepatocellular carcinoma (HCC). NAFLD is a kind of metabolic disordered disease, which is commonly associated with lipid metabolism, insulin resistance, oxidative stress, inflammation, and fibrogenesis, as well as autophagy. Growing studies have shown Notch signaling pathway plays a pivotal role in the regulation of NAFLD progression. Here, we review the profile of the Notch signaling pathway, new evidence of Notch signaling involvement in NAFLD, and describe the potential of Notch as a biomarker and therapeutic target for NAFLD treatment.

## Introduction

Non-alcoholic fatty liver disease (NAFLD), affecting over a quarter of the global population, has emerged as the highest prevalent type of chronic liver disease (Younossi et al., 2016). NAFLD encompasses a spectrum of progressive liver diseases including simple steatosis (SS), non-alcoholic fatty steatohepatitis (NASH), fibrosis, cirrhosis, and hepatocellular carcinoma (HCC) (D. Q. Huang et al., 2021; Powell et al., 2021). NAFLD is defined by the presence of steatosis in >5% of hepatocytes in histological analysis and exclusion of excessive alcohol consumption daily (≥30 g for men and ≥20 g for women) (EASL et al., 2016). Evidence suggests that NAFLD is related to liver manifestations of metabolic syndrome such as obesity, diabetes, insulin resistance (IR), and dyslipidemia (Younossi et al., 2018; Jarvis et al., 2020).

The individual clinical outcomes of patients with NAFLD are highly variable. For the majority of patients with simple steatosis, their liver disease is in non- or slow-progression. A prospective cohort study reported in a three-year period, over 20% of patients with simple steatosis developed into NASH (Wong et al., 2010), a more severe stage in which fatty liver is accompanied by necroinflammatory changes like hepatocyte ballooning and lobular inflammation (Vernon et al., 2011). In the final stages, collagen deposition and subsequent vascular remodeling result in fibrosis and cirrhosis (EASL et al., 2016). Thus far, there is no accurate non-invasive diagnostic biomarker and effective treatment toward NAFLD (Francque and Vonghia, 2019; Younossi, 2019), and current therapy is mainly focused on lifestyle changes (EASL et al., 2016; Chalasani et al., 2018).

**FIGURE 1:**
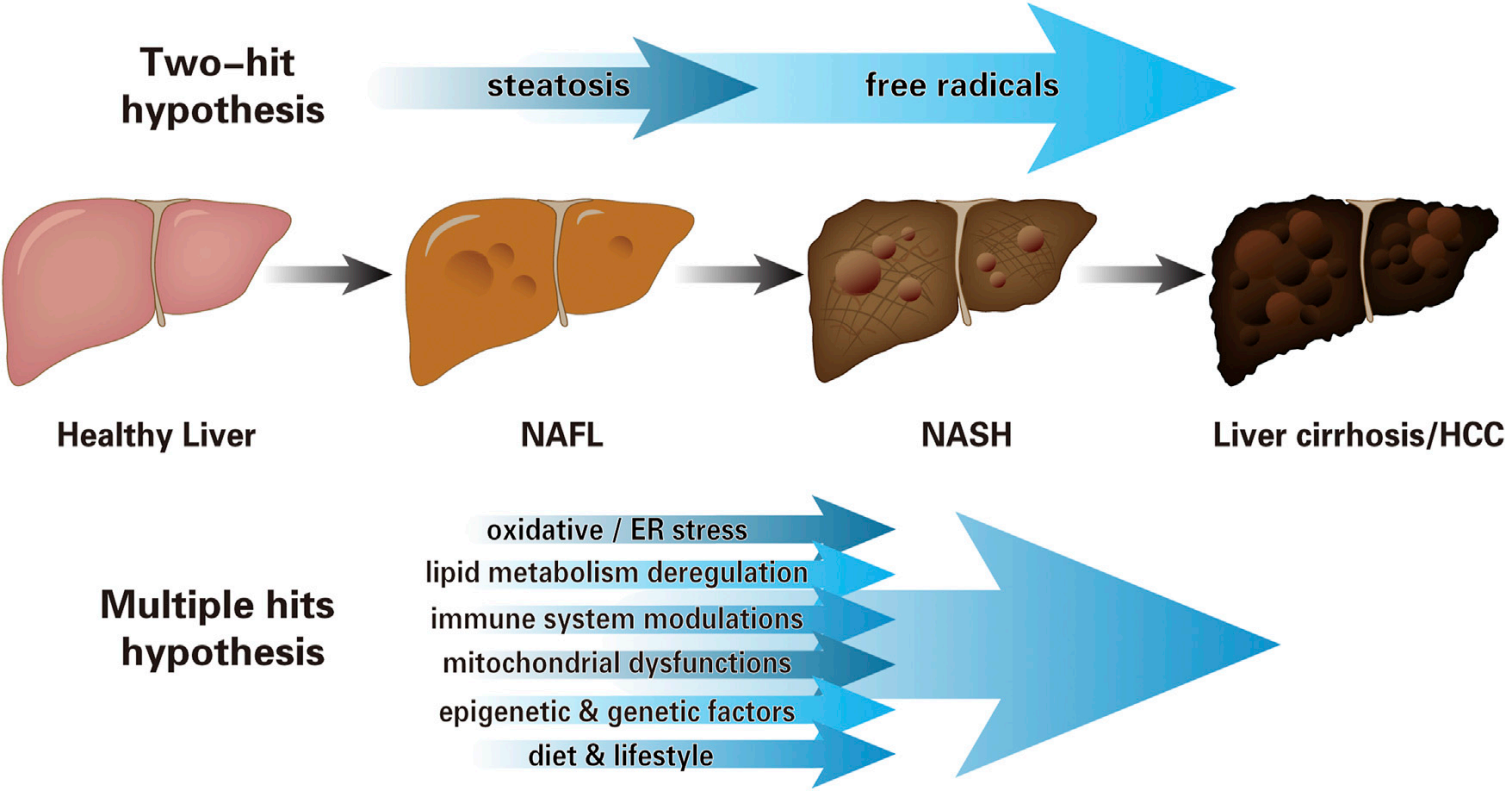
The pathogenesis of NAFLD. Schematic representation illustrating cognition toward the progression of NAFLD: from “two-hit” hypothesis to “multiple hits” hypothesis. The early “two-hit” hypothesis, in which the first “hit” is steatosis, leads to the second “hit”: oxidative stress, endotoxin, etc. The “multiple hits” hypothesis considers several parallel hits jointly affect the NAFLD pathogenesis, which includes, but is not limited to oxidative and/or ER stress, lipid metabolism deregulation, immune system modulations, mitochondrial dysfunctions, lifestyle, and epigenetic and genetic factors (NAFL, non-alcoholic fatty liver, i.e., simple steatosis without hepatocellular injury; NASH, non-alcoholic steatohepatitis; HCC, hepatocellular carcinoma; ER, endoplasmic reticulum).

Studies have shown that NAFLD is mainly characterized by hepatocyte inflammation and steatosis in the early stage and fibrosis and/or cirrhosis in the late stage (Wang et al., 2020). However, the pathogenesis of NAFLD has not been fully understood. In 1998, scientists first proposed the “two-hit” hypothesis to explain that steatosis (the first “hit”) and other factors associated with free radicals (the second ‘‘hit”) are necessary for NASH progression (Day and James, 1998). In recent years, based on animal models and descriptive clinical trials, the “multiple hits” hypothesis is widely accepted (Tilg and Moschen, 2010; Buzzetti et al., 2016; Tilg et al., 2021). The primary hit is the infiltration and pro-inflammatory state of macrophages in the visceral adipose tissue, resulting in IR. Meanwhile, the abnormal lipolysis increases the delivery of fatty acids to the liver, and along with steatosis, aggravates the lipid metabolic burden. The imbalance results in the formation of lipotoxic lipids that generate a series of multiple hits, including oxidative and/or endoplasmic reticulum (ER) stress, inflammasome activation, and apoptotic damage, followed by inflammation, tissue regeneration, and fibrogenesis (Tilg and Moschen, 2010; Bessone et al., 2019; Sanyal, 2019). Besides, mitochondrial dysfunctions, lifestyle, and epigenetic and genetic factors also jointly affect the occurrence and progression of the NAFLD (Loomba et al., 2021) (Figure 1).

Notch signaling pathway plays a crucial role in cell differentiation (Amsen et al., 2009; Amsen et al., 2015), proliferation (Bartolome et al., 2019), and apoptosis (Guruharsha et al., 2012). Recently, it has also been demonstrated that Notch is involved in liver development, homeostasis, and metabolism (Bi and Kuang, 2015; Geisler and Strazzabosco, 2015; Adams and Jafar-Nejad, 2019). However, the association of the Notch signaling with NAFLD has rarely been reported. Here we review the recent advances in Notch signaling in liver pathophysiology and analyze the Notch signaling pathway as a potential target to prevent and treat NAFLD.

## Overview of Notch Signaling Pathway

Notch signaling is a juxtracrine signal transduction mechanism that enables cell-cell communication directly (Artavanis-Tsakonas et al., 1999). In mammals, four receptors (Notch1-4) and five ligands [Jagged (JAG) 1-2, Delta-like ligand (DLL) 1, 3, and 4] have been identified in canonical Notch signaling (D'Souza et al., 2010). In the liver of adults, four Notch receptors are expressed, while only two Notch ligands (JAG1 and DLL4) are expressed (Y. Chen et al., 2012). The ligand-receptor interaction is the initiation of Notch signaling pathway, making various cellular regulations more precise and orderly (Bray, 2016).

**FIGURE 2:**
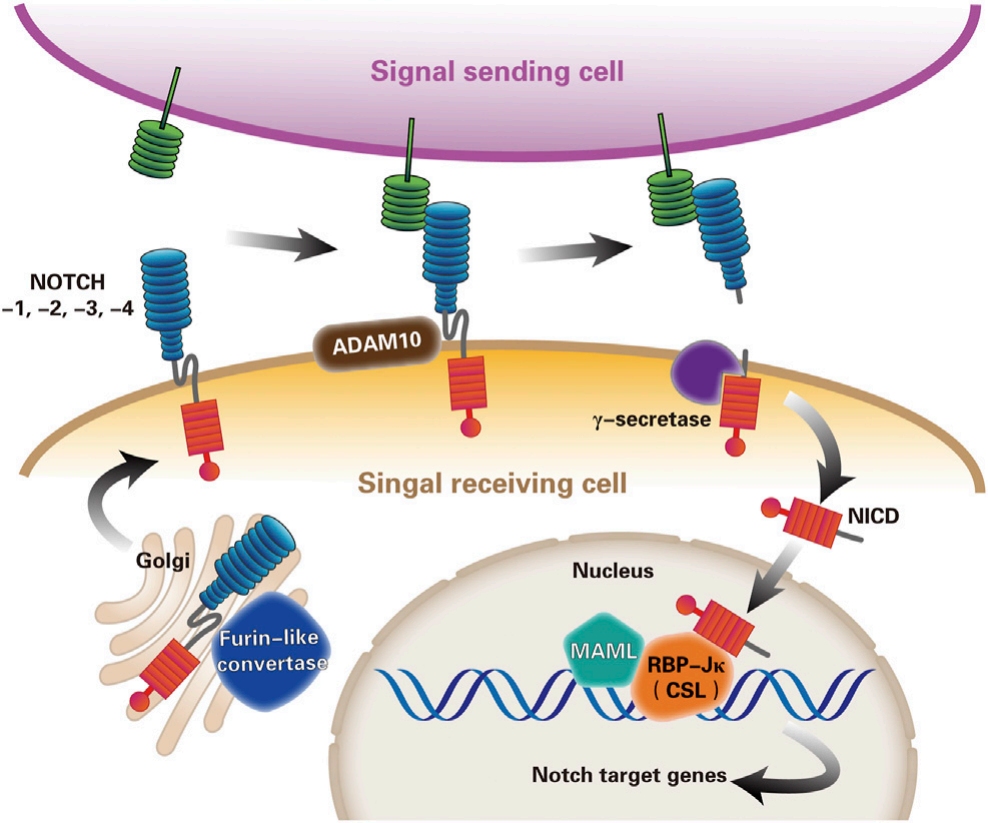
The canonical Notch signaling pathway. Notch signaling pathway is currently thought to be activated by three steps of proteolysis. First, the mammalian Notch receptors are cleaved by a furin-like convertase in the Golgi compartment. After digestion, the extracellular subunits and transmembrane subunits formed by Ca^2+^ dependent non-covalent bonding to form heterodimers, and exocytosed to the cell membrane become mature Notch receptors. Second, Notch ligand-receptor binding enables proteolytic cleavage of the Notch extracellular domain by ADAM10 metalloprotease, and Notch receptor releases extracellular subunits. Third, γ-secretase complex cleaves the remnant receptor to allow the release and nuclear translocation of the NICD, where NICD forms a trimeric complex with transcription factor RBP-Jκ(or CSL) and the co-activator MAML, imitating the expression of Notch target genes transcription. (ADAM, a disintegrin and metalloprotease10; RBP-Jκ, recombination signal binding protein immunoglobulin kappa J; NICD, Notch intracellular domain; CSL, CBF1–suppressor of hairless–LAG1; MAML, mastermind-like).

The core signaling pathway most commonly used to describe Notch-dependent processes is named the canonical Notch signaling pathway (Andersson et al., 2011; Guruharsha et al., 2012). The ligand presented by the Notch signal sending cell binds to the receptor on the signal-receiving cell. The endocytosis of the ligand leads to a conformational change of the Notch receptor, exposing the cleavage site of the ADAM10. Subsequent cleavage of the γ-secretase complex releases the Notch intracellular domain (NICD) (Kopan and Ilagan, 2009). NICD then migrates to the nucleus, binds to the transcription factor RBP-Jκ (also called CSL) (Kovall and Blacklow, 2010), and recruits the co-activator Mastermind-like (MAML) to initiate downstream gene transcription, including the hairy enhancer of split (*HES*) and *HES*-related (*HEY*) family genes (Nam et al., 2006; Wilson and Kovall, 2006; Bolos et al., 2007; Guruharsha et al., 2012) (Figure 2).

Different from other classical signal transduction processes, the canonical Notch signaling pathway is characterized by the lack of cascade amplification in the transduction process, and only NICD is generated after a Notch receptor is consumed. Therefore, its signal intensity is crucial for generating the corresponding cellular response, and any deviation in the expression level of any molecular component in the Notch signaling pathway may have a vital impact (Andersson et al., 2011). For example, Alagille syndrome (AGS) is caused by mutations in the gene for the Notch ligand JAG1 and NOTCH2 receptor (McDaniell et al., 2006). Currently, various Notch signaling pathway modulation approaches have been explored, including inhibition of the ligand-receptor interaction and interference with the proteolytic process of the receptor (Groth and Fortini, 2012; Shao et al., 2012; Andersson and Lendahl, 2014). Several Notch inhibitors are demonstrated effects in the NAFLD (Table 1).

**TABLE 1:** Major Notch signaling inhibitors in NAFLD

Inhibitor	Target	Function	Object	References
Peroxiredoxin 6 (PRDX6)	*Notch1*	Improve lipid accumulation through induction of mitophagy	Mice	Lee et al. (2019)
Notch1 decoy	*Notch1*	Decrease hepatic glucose production	Mice	Funahashi et al. (2008) Pajvani et al. (2013)
N-[N-(3,5-Difluorophenacetyl)-L-alanyl]-S-phenylglycine t-butyl ester (DAPT)	*Notch1*	Alleviate lipid accumulation and hepatocyte injury	Mice	Zhang et al. (2021)
Delta-like1 homolog (DLK1)	*Notch1*	Reduce hepatic steatosis and improve glucose and insulin tolerance	Mice	Lee et al. (2016)
Triptolide (TP)	*Notch1*	Initiate oxidative stress in hepatocyte	Mice	Shen et al. (2019)
Hepatocyte Toll-like receptor 4 (TLR4)	*Jag1/JAG1*	Reduce NASH related liver fibrosis	Mice/Human	Yu et al. (2021)
Nuclear factor (erythroid-derived 2)-like 2 (Nrf2)	NICD	Ameliorate hepatic lipogenesis dyslipidemia and insulin resistance	Mice	Chartoumpekis et al. (2018)
γ-secretase inhibitor (GSI)	γ-secretase	Improve glucose metabolism and ameliorate liver fibrosis	Mice	Richter et al. (2020)
Liver-specific *Rbp-jκ* knockout (L-*RBP-Jκ*)	*RBP-Jκ*	Protect from obesity-induced insulin resistance	Mice	Pajvani et al. (2011)
Silybin (SIL)	*NOTCH1*	Hepatoprotective and antitumorigenic effect in HCC cells	Human	Zhang et al. (2013)
Delta-tocotrienol (δ-T)	*NOTCH1*	Reduce biochemical markers of hepatocellular injury and steatosis	Human	Pervez et al. (2020)

## Notch in Lipid Metabolism

As the central hub of lipid homeostasis, the liver is responsible for coordinating the whole process of lipid circulation, including the synthesis, export, redistribution, and utilization of free fatty acids (Nguyen et al., 2008). The main pathways that constitute hepatic lipid homeostasis, including uptake of circulating lipids, *de novo* lipogenesis (DNL), fatty acid oxidation (FAO), and export as very-low-density lipoprotein (VLDL) particles (Gluchowski et al., 2017; Ipsen et al., 2018). Hallmarked by hepatic steatosis, NAFLD is connected with lipid metabolism. When lipid acquisition exceeds lipid disposal in the liver, that is, the uptake of fatty acids and DNL covering oxidation and output of fatty acids, hepatic steatosis occurred (Ipsen et al., 2018). Feng et al. (2017) proposed that no significant differences between free fatty acids (FFAs) in lean or obese patients with NAFLD were observed, and the value of serum FFAs in early diagnosis of NAFLD.

The studies suggested that nutrition-induced activation of mammalian target of rapamycin (mTOR) may cause an increase in liver lipid content, which also increases the activity of basal serine/threonine kinases, leading to a self-perpetuating lipogenic cycle (Lamming and Sabatini, 2013; Caron et al., 2015; Han and Wang, 2018). Pajvani et al*.* (Pajvani et al., 2013) demonstrated that inhibition of Notch signaling prevented hepatic steatosis by blocking mTOR complex 1 (mTORC1) activity, which could be reversed by rapamycin treatment. They also showed that Notch signaling augmented mTORC1 function and SREBP1c-mediated lipogenesis and that inhibition of hepatic Notch signaling protects from the fatty liver by reducing DNL.

Although the specific pathogenesis of lipid metabolism disorder in NAFLD patients is still not completely clear, studies have shown it may be associated with Notch pathway regulation (Li et al., 2019). Ding et al. (2020) investigated the dynamic role of Notch gene expression in the development of NAFLD *in vitro* and *in vivo*. They used palmitic acid (PA) and methionine-choline-deficient (MCD) models to assess notch signaling genes expression changes at different time points. Based on the characteristics of *Notch* mRNA expression levels, they evaluated that expression of *Notch3* mRNA has been dynamically changed significantly in the development of hepatic steatosis during NAFLD (Ding et al., 2020). Furthermore, Auguet et al. (2020) explored the association between the *Notch* transcriptional repressor and hepatic expression of lipid metabolism-related genes in a cohort of women with NAFLD. They found a negative relationship between hepatic *HEY2* expression and low-density lipoprotein (LDL) cholesterol (Auguet et al., 2020).

## Notch in Insulin Resistance

It is generally recognized that IR is pivotal in the pathogenesis and progression of NAFLD (Lomonaco et al., 2012). IR is essentially a decrease in the sensitivity of whole-body, liver, and adipose tissue to insulin, which is involved in the development of hepatic steatosis (E. Bugianesi, 2010). In NAFLD patients, increases in circulating glucose and insulin associated with IR promote hepatic DNL (Smith et al., 2020). Specifically, when IR occurs, it causes an impaired ability of insulin to inhibit adipose tissue lipolysis, resulting in increased delivery of FFAs to the liver (Bugianesi et al., 2005). Meanwhile, large lipid deposition promotes IR, which leads to fasting hyperglycemia and compensatory hyperinsulinemia, further contributing to the pathophysiology of NAFLD via exacerbating DNL (Donnelly et al., 2005).

The abnormal activation of Notch signaling pathway and IR are closely linked. It is recognized factor forkhead box protein O1 (FOXO1) has a beneficial effect on insulin-mediated glucose homeostasis (Matsumoto et al., 2007; O-Sullivan et al., 2015). Notch signal mainly affects hepatic glucose via the synergistic effect of NICD and FoxO1 transcription. Glucose-6-phosphatase catalytic subunit (G6PC) and phosphoenolpyruvate carboxykinase (PCK1) are both rate-limiting enzymes of hepatic glycogenolysis and gluconeogenesis, which would be correlated with Notch activation (Valenti et al., 2013; Dongiovanni et al., 2016). Pajvani et al. (2011) reported that combined haploinsufficiency of FoxO1 and Notch1 notably improves insulin sensitivity in diet-induced IR. Hepatic overexpression of Notch1 regulates hepatic gluconeogenesis by inducing G6PC in a FoxO1-dependent mode, in turn, aggravates insulin resistance (Pajvani et al., 2011; Bernsmeier et al., 2016). Additionally, the reduction of metabolic activity in brown adipose tissue (BAT) has been found connected with IR in human (Stanford et al., 2013; Mottillo et al., 2016). Bi et al. (2014) revealed mice in which *Notch1* or *Rbp-jκ* selectively deleted in adipocytes show upregulated expression of BAT-specific genes and improvement in glucose tolerance and insulin sensitivity.

Based on the close relation between IR and Notch, several possible pharmacological targets of NAFLD are identified. Blocking the abnormal expression of Notch at the gene level can inhibit the accumulation of liver gluconeogenesis and triglycerides (TGs), thereby reducing the risk of NAFLD. The cleavage of NICD by γ-secretase inhibitor (GSI) exhibited an improvement of glucose homeostasis and insulin sensitivity in diet-induced obese (DIO) mice (Pajvani et al., 2011). Lee et al. (2016) demonstrated that Delta-like 1 homolog (DLK1), an inhibitory regulator of Notch signaling, would reduce hepatic steatosis and hyperglycemia via exogenous administration. Chartoumpekis et al*.* (2018) showed nuclear factor (erythroid-derived 2)-like 2 (Nrf2) could profoundly ameliorate hepatic lipogenesis and IR by repressing NICD. Besides, researchers also found plant extracts (such as curcumin) have been shown to suppress NOTCH1, which could ameliorate fatty liver and enhance insulin sensitivity in the high-fat diet (HFD) model (Zhao et al., 2017; Saadati et al., 2019; El et al., 2021).

## Notch in Oxidative Stress

Oxidative stress (OS) is a concept used to describe an imbalance between pro-oxidants and antioxidants, leading to cellular damage and tissue injury (Sies, 2015). The chronic high-calorie diet causes lipid accumulation in hepatocytes and excessive generation of reactive oxygen species (ROS) (Sahini and Borlak, 2014). Meanwhile, affected by lipotoxicity from high levels of lipid metabolites, OS inhibits insulin sensitivity and facilitates DNL (Gehrke and Schattenberg, 2020).

In the pathophysiological process of NAFLD, OS is considered a pivotal mediator of the inflammatory response (Koek et al., 2011). Notch signaling has been reported to be associated with steatosis and OS. It has been proposed that ROS like H_2_O_2_ regulates the expression of Notch (Marinho et al., 2014). Notch1 regulates the expression of lipid oxidation genes and exhibited an obvious lipid accumulation reduction in Notch1 deficient antisense transgenic (NAS) mice (Song et al., 2016). Similarly, Notch1 inhibitor reduces ethanol-induced OS and lipid accumulation in HepG2 cells (Wang et al., 2014).

Among the multiple mechanisms that accelerate the progression of NAFLD to NASH, mitochondrial dysfunction is the prime one (Caldwell et al., 1999). Mitochondrial abnormalities disrupt the balance between pro-oxidants and antioxidants, leading to an increase of FFAs (Begriche et al., 2013). Peroxiredoxin 6 (PRDX6) is a mitochondrial antioxidant enzyme and is highly expressed in the liver (Fisher, 2011; Arriga et al., 2019). Lee et al. (2019) demonstrated that PRDX6 induces effects of maintaining mitochondrial integrity and inhibits OS-induced Notch signaling, thereby reducing ROS production and lipid accumulation. They pointed out that PRDX6 mitophagy-mediated mechanisms offer endogenous protection against NAFLD (Lee et al., 2019).

Moreover, triptolide (TP) is the main ingredient of the medicinal herb Tripterygium wilfordii Hook f (TWHF) (Ziaei and Halaby, 2016). TP caused hepatotoxicity through initiating OS. Shen et al. (2019) investigated TP inhibited the protein expression of Notch1 and NICD, and the activation of Notch signaling has the potential to protect against TP-induced live injury. Interestingly, Huang et al. (2021) demonstrated that dose-related TP as an allosteric AMPK agonist alleviates NAFLD. Combined, the regulation of Notch signaling pathway may better enable TP to play a protective role in NAFLD.

## Notch in Inflammation and Fibrogenesis

Liver fibrosis is a decisive factor of liver disease progression, particularly as it is associated with adverse prognosis and mortality in patients with NASH (Vilar-Gomez et al., 2018; Powell et al., 2021). Even in the early stage of fibrosis, it is shown a series of adverse liver-related events are gradually increasing (Angulo et al., 2015; Dulai et al., 2017; Hagstrom et al., 2017). In advanced NASH, hepatocytes are partially replaced by fibrotic scar tissue, the severe pathological change makes it difficult to treat NASH by correcting the underlying metabolic abnormality. Therefore, anti-fibrosis has become the focus of NASH therapy.

Notch activity is almost absent in healthy adult hepatocytes, mildly elevated in simple steatosis, and significantly increased in NASH (Valenti et al., 2013; Zhu et al., 2018). In various mouse models of fibrosis, over 80% of collagenous myofibroblasts are caused by hepatic stellate cell (HSC) (Mederacke et al., 2013). Notch-activated hepatocytes facilitate liver profibrogenic in NASH by both osteopontin (Opn) secretion mediated HSC activation *in vitro* and *in vivo* (Zhu et al., 2018), leading to a continuous extracellular matrix (ECM) accumulation and liver parenchyma gradually replaced by fibrous tissue (Mederacke et al., 2013). Conversely, in Notch loss-of-function mouse models, hepatocyte-specific liver inflammation and fibrosis are reduced, suggesting maladaptive hepatocytic Notch response to NASH-associated liver fibrosis (Zhu et al., 2018).

Sawitza et al. (2009) explored Jag1 as one of the cell surface ligands in Notch signaling activates HSC to stimulate α-SMA and collagen production. Yu et al. (2021) proved increasing Jag1 is responsible for fibrosis-inducing Notch reactivation. Also, other hepatic non-parenchymal cells could activate the Notch pathway to promote NASH latently through various mechanisms. Duan et al. (2018) investigated Notch activation in liver sinusoids endothelial cell (LSEC), which leads to HSC activation and the subsequent hepatic fibrosis, by downregulating eNOS-sGC signaling. Besides, researchers found that inhibitors inactivate M1 polarization of macrophage by regulating Notch signaling could reduce the secretion of inflammatory cytokine and fibrogenesis in CCl_4_-induced liver injury mice (Bansal et al., 2015; Xu et al., 2015; Sheng et al., 2020). Additionally, γ-secretase inhibitor (Chen et al., 2012) and Notch3 siRNA (Y. X. Chen et al., 2012) suppressed the myofibroblastic gene expression of rat HSC line by blocking Notch signaling. Therefore, selective interruption of these Notch-related targets may provide more anti-fibrosis strategies for NAFLD (Romeo, 2019).

## Notch in Autophagy

Autophagy is a process in which cells degrade and metabolize their own damaged organelles or protein aggregation (Wang et al., 2019), which plays a vital role in regulating multiple liver functions and maintaining hepatic homeostasis (Ueno and Komatsu, 2017). Accumulating evidence suggests autophagy regulates liver-mediated systemic glucose and lipid metabolism (Singh et al., 2009; Galluzzi et al., 2014). Meanwhile, the liver is surrounded by exogenous substances from the portal vein circulation, including potential inflammatory mediators, in which autophagy has major cell-protective and anti-inflammatory effects (Deretic et al., 2013; Deretic and Levine, 2018; Hazari et al., 2020). All of the above suggests autophagy is associated with the occurrence and development of various liver diseases such as NAFLD.

The lipid droplets (LDs) are specialized cytosolic organelles in which some organs including the liver store neutral lipids (such as TGs) to protect from lipotoxicity (Gross and Silver, 2014). The progression of LDs degradation is regarded as a specific form of autophagy, also known as lipophagy (Garcia et al., 2018). Recent studies have revealed that disturbances in lipophagy have been linked to hepatic lipid accumulation, the process of lipophagy could be regarded as a new way of controlling NAFLD development (Grefhorst et al., 2021).

Because autophagy can remove damaged organelles, autophagy may alleviate hepatocellular injury during NASH. The protective effects of carbamazepine-induced autophagy could reduce steatosis and improve IR in the NAFLD model (Lin et al., 2013). Indeed, modulating autophagy may prevent the progression of NAFLD. Zhang et al. (2021) investigated that *Notch1* is an activated intensity of autophagy in FFA-treated HepG2 cells, and decreased *Notch1* levels may alleviate hepatocyte damage by enhancing autophagy, which could be reversed by autophagy inhibitor chloroquine. Niture et al*.* (2018) demonstrated that inhibition of Notch reduced the expression of autophagy biomarker and serotonin-mediated liver cell steatosis. These findings provide helpful clues for the strategy of Notch signaling pathway to regulate autophagy and thereby remit the progression of NAFLD.

## Notch in NAFLD-Related HCC

HCC is the fourth leading cause of cancer-related deaths worldwide and occurs in patients with various chronic liver diseases (Bray et al., 2018; Llovet et al., 2021). Although hepatitis B virus (HBV) infection has been the prominent risk factor of HCC, NAFLD has become the most rapidly growing driver of HCC in many countries (Younossi et al., 2019; Hester et al., 2020). The incidence in patients with NAFLD-related HCC increases with the histological stage, which is highest in patients with NAFLD-cirrhosis (Ioannou, 2021).

Thus far, the exact pathogenesis underlying NAFLD-induced HCC is only incompletely understood but mainly focuses on the effects of DNA damage response, inflammation, autophagy, and intestinal microbiota (Anstee et al., 2019; Behary et al., 2021). In addition, the chronic activation of metabolic pathways seems to play a critical role (Baffy et al., 2012). These pathways may provoke infinite hepatocyte proliferation and genomic instability, and on the other hand, provide a microenvironment conducive to malignant transformation and tumor growth.

Recent studies suggest that Notch signaling pathway is frequently associated with tumorigenesis (Nowell and Radtke, 2017). Selective blocking of Notch1 inhibits cancer cell growth and deregulates angiogenesis (Wu et al., 2010). By performing RNA sequencing of hepatocyte populations HFD-fed reporter mice, Zhu et al. (2021) illustrated that Notch-active hepatocytes showed transcriptional enrichment of ECM-related genes, which may represent a mechanism that persists in the tumorigenic process. Furthermore, they found HFD-diet mice with Notch-active mutation spontaneously formed fully developed liver tumors (Zhu et al., 2021). Therefore, it can be inferred that the continuous activation of Notch signaling pathway promotes the occurrence of NAFLD-related HCC.

## Conclusion and Perspective

NAFLD is a manifestation of metabolic syndrome in the liver. With the changes in lifestyle and dietary habits, the incidence of NAFLD is rising rapidly. The previous studies have revealed the significance of the Notch signaling pathway in metabolism. The abnormal expression of Notch may lead to several metabolic disorders, thus inducing NAFLD. Although the relation between NAFLD and Notch signaling has been observed both *in vitro* and *in vivo*, most of the research findings are based on phenotypic studies and the underlying mechanisms and potential associations between different Notch molecules, and require further in-depth research.

**FIGURE 3:**
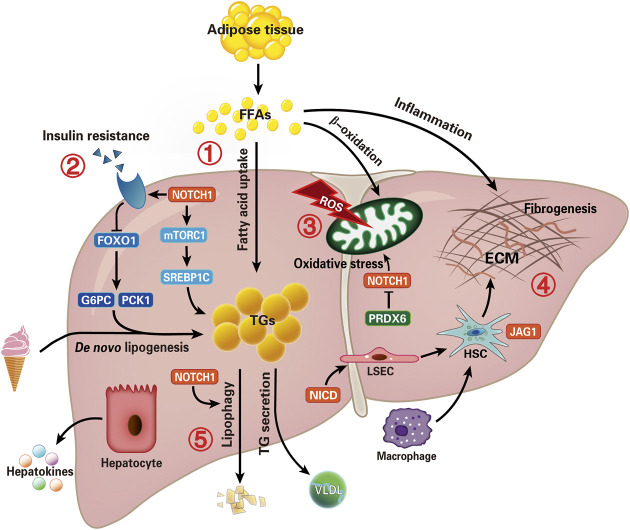
Overview of potential therapeutic targets of Notch signal pathway in NAFLD. Summarize the recent critical advances evolving Notch signaling in the NAFLD. Intrahepatic lipid levels depend on the balance between lipid acquisition and disposal. Therefore, lipid accumulation is the result of uptake of fatty acids and *de novo* lipogenesis exceeding export as VLDL and oxidation of fatty acid, which is also a main pathophysiological change of NAFLD. The five potential pathways to intervene in NAFLD are focused on (1) hepatic lipid accumulation, (2) insulin resistance, (3) oxidative stress, (4) inflammation and fibrogenesis, and (5) autophagy (lipophagy) progression. Although several aspects of these functions remain to be fully understood, these findings offer an intriguing rationale for investigating Notch-based therapies in patients with NAFLD. Furthermore, the “hepatokines” secreted by hepatocytes may help reveal the complex molecular regulation in NAFLD. (NICD, Notch intracellular domain; FFAs, free fatty acids; mTORC1, mammalian target of rapamycin complex one; FOXO1, factor forkhead box protein O1; G6PC, glucose-6-phosphatase catalytic subunit; PCK1, phosphoenolpyruvate carboxy kinase; TGs, triglycerides; VLDL, very-low-density lipoprotein; ROS, reactive oxygen species; PRDX6, peroxiredoxin 6; ECM, extracellular matrix; HSC, hepatic stellate cell; LSEC, liver sinusoids endothelial cell).

The development of liver-specific Notch inhibitors is pivotal for the treatment of NAFLD-related hepatic lipid accumulation, IR, OS, fibrogenesis, and autophagy progression (Figure 3). But until now, most intervention studies are conducted in animal models (especially mice), the potential role of Notch regulators in human NAFLD needs to be explored extensively. Recently, the rising field of “hepatokines” biology would help reveal the complex molecular regulation in NAFLD (Watt et al., 2019). If so, it would promote the development of more non-invasive diagnostic tests to improve early diagnosis rates.

There is no specific and effective pharmacotherapy toward NAFLD, however, some drugs have shown therapeutic potential by regulating a Notch signal pathway. Vitamin E (α-tocopherol) is a dietary antioxidant recommended as a treatment for NASH (Yakaryilmaz et al., 2007; Chalasani et al., 2018). Recent clinical research supports vitamin E use brought obvious histological benefits and improved prognosis in patients with NASH (Sato et al., 2015; Brunt et al., 2019; Vilar-Gomez et al., 2020). δ-tocotrienol, an isomer of vitamin E, has been explored to inhibit tumor invasion and metastasis via downregulating the NOTCH1 signaling pathway (Rajasinghe et al., 2018). Notably, Pervez et al. (2020) launched a randomized, double-blind, placebo-controlled trial of 71 patients with NAFLD. Compared with placebo, δ-tocotrienol significantly reduced biochemical markers of hepatocellular injury and steatosis in patients (Pervez et al., 2020). Silybin (SIL), a hepatoprotective drug, could be an inhibitor targeting the NICD, RBP-Jκ, and Hes1 proteins in HCC cells and exert antitumorigenic effects (Zhang et al., 2013).

The precise drug delivery without toxicity brings a wide application prospect for the treatment of NAFLD. A nanoparticle-mediated delivery system to target GSI in the liver (GSI NPs) has been developed (Richter et al., 2020), which avoids goblet cell metaplasia caused by intestinal Notch inhibition (van Es et al., 2005). Based on similar studies above would advance clinical therapy research, thereby optimizing therapies for various NAFLD subtypes to increase the cure rate while complications can be decreased. In a word, findings on Notch signaling pathway research could bring NAFLD patients a hopeful future with ever more promising targets for prevention and treatment.

## Glossary

AGS: Alagille syndrome
DIO: diet-induced obese
DLK1: Delta-like 1 homolog
DNL: de novo lipogenesis
ECM: extracellular matrix
ER: endoplasmic reticulum
FAO: fatty acid oxidation
FFAs: free fatty acids
FOXO1: factor forkhead box protein O1
G6PC: glucose-6-phosphatase catalytic subunit
GSI: γ-secretase inhibitor
HCC: hepatocellular carcinoma
HFD: high-fat diet
HSC: hepatic stellate cell
IR: insulin resistance: insulin resistance
IR: insulin resistance: insulin resistance
LDs: lipid droplets
LSEC: liver sinusoids endothelial cell
MCD: methionine-choline-deficient
mTOR: mammalian target of rapamycin
mTORC1: mTOR complex 1
NAFL: non-alcoholic fatty liver
NAFLD: non-alcoholic fatty liver disease
NASH: non-alcoholic fatty steatohepatitis
NAS: Notch1 deficient antisense transgenic
NICD: Notch intracellular domain
Nrf2: nuclear factor (erythroid-derived 2)-like 2
Opn: osteopontin
OS: oxidative stress
PA: palmitic acid
PCK1: phosphoenolpyruvate carboxy kinase
PRDX6: Peroxiredoxin 6
ROS: reactive oxygen species
SIL: Silybin; δ-T(Delta-tocotrienol)
SS: simple steatosis
TGs: triglycerides
TP: triptolide
TWHF: tripterygium wilfordii Hook f
VLDL: very-low-density lipoprotein
